# Equine Activity Time Budgets: The Effect of Housing and Management Conditions on Geriatric Horses and Horses with Chronic Orthopaedic Disease

**DOI:** 10.3390/ani11071867

**Published:** 2021-06-23

**Authors:** Zsofia Kelemen, Herwig Grimm, Claus Vogl, Mariessa Long, Jessika M. V. Cavalleri, Ulrike Auer, Florien Jenner

**Affiliations:** 1 Equine Surgery Unit, University Equine Hospital, Department of Companion Animals and Horses, University of Veterinary Medicine Vienna, Veterinaerplatz 1, 1210 Vienna, Austria; Zsofia.Kelemen@vetmeduni.ac.at; 2 Unit of Ethics and Human-Animal-Studies, Messerli Research Institute, University of Veterinary Medicine Vienna, Medical University of Vienna, University of Vienna, Veterinaerplatz 1, 1210 Vienna, Austria; Herwig.Grimm@vetmeduni.ac.at (H.G.); Mariessa.Long@vetmeduni.ac.at (M.L.); 3 Department of Biomedical Sciences, Institute of Animal Breeding and Genetics, University of Veterinary Medicine Vienna, Veterinaerplatz 1, 1210 Vienna, Austria; Claus.Vogl@vetmeduni.ac.at; 4 Equine Internal Medicine Unit, University Equine Hospital, Department of Companion Animals and Horses, University of Veterinary Medicine Vienna, Veterinaerplatz 1, 1210 Vienna, Austria; Jessika.Cavalleri@vetmeduni.ac.at; 5 Anaesthesiology and Perioperative Intensive Care Medicine Unit, Department of Companion Animals and Horses, University of Veterinary Medicine Vienna, Veterinaerplatz 1, 1210 Vienna, Austria

**Keywords:** horse, equine, activity, time budget, behaviour

## Abstract

**Simple Summary:**

Housing and management conditions strongly influence the health, welfare and activity behaviour of horses. To improve horses’ living conditions, it is necessary to establish objective and quantifiable measures that allow for a comparison between environmental living conditions and of how horses of different ages and health statuses are influenced by these environmental conditions. Thus, the aim of the present study was to record and compare time budgets (=percentage of time spent on specific activities) of old (≥20 years) horses and of horses suffering from chronic orthopaedic disease that are living in different husbandry conditions with an automated tracking device. These horses were found to spend similar percentages of time feeding, resting and moving compared to healthy controls. Horses living on different farms and with different turn-out conditions differed in their time budgets. Horses living in open-air group housing on a paddock had less pronounced peaks in their feeding and movement activities over time compared to horses living in more restricted husbandry systems. The findings of the study can help to identify potential improvements of husbandry conditions of horses to maximise their health and welfare.

**Abstract:**

Housing and management conditions strongly influence the health, welfare and behaviour of horses. Consequently, objective and quantifiable comparisons between domestic environments and their influence on different equine demographics are needed to establish evidence-based criteria to assess and optimize horse welfare. Therefore, the present study aimed to measure and compare the time budgets (=percentage of time spent on specific activities) of horses with chronic orthopaedic disease and geriatric (≥20 years) horses living in different husbandry systems using an automated tracking device. Horses spent 42% (range 38.3–44.8%) of their day eating, 39% (range 36.87–44.9%) resting, and 19% (range 17–20.4%) in movement, demonstrating that geriatric horses and horses suffering from chronic orthopaedic disease can exhibit behaviour time budgets equivalent to healthy controls. Time budget analysis revealed significant differences between farms, turn-out conditions and time of day, and could identify potential areas for improvement. Horses living in open-air group housing on a paddock had a more uniform temporal distribution of feeding and movement activities with less pronounced peaks compared to horses living in more restricted husbandry systems.

## 1. Introduction

Domestic horses are kept in a variety of housing systems, which offer differing levels of physical freedom, foraging opportunities and contact with conspecifics [1,2,3,4,5,6]. The effect of different housing systems on equine welfare and behaviour, however, remains understudied and research to date has been largely limited to small groups of healthy horses and manual scoring of behaviour. As housing and management conditions have been shown to strongly influence the health, welfare and activity behaviour of horses [1,2,3,4,5,6,7,8,9], comparing the time budget (=percentage of time spent on specific activities) of horses in different domestic environments may help to objectively and quantitatively assess welfare and monitor interventions. In this context, it is important to also study horses at risk of poor welfare, such as horses with chronic diseases or geriatric animals, to establish the time budget parameters for this equine demographic.

Changes in horses’ time budgets may reflect a coping mechanism to an inappropriate environment or health issues and indicate a potential welfare impairment [10,11,12,13]. The time budget of feral, free-ranging horses is typically used as a baseline for comparison with the view that optimal welfare is reflected in wild-type behaviour [7,10,11,12,13,14,15,16,17,18]. However, the validity of such comparisons has not been assessed and the environment of most domestic horses is far removed from the wild, which rarely meets the five freedoms of animal welfare [3,4,11,14,15,19,20,21,22]. Free-ranging horses roam areas of land up to 78 km^2^ with the size of the home range depending on resource availability and spend a large portion of their time budget (50–67%, 12–16 h) grazing [16,23,24,25,26,27]. Domestic horses, in contrast, are typically confined to small stables or paddocks and have restricted access to roughage, which entails possible threats to equine welfare and health [22]. While the constrained environment meets a horse’s basic resource requirements, such as food and shelter, it may present challenges to instinctive and innate behaviour patterns by removing horses from exposure to their natural environmental stimuli, including the continuous social foraging, feeding and low intensity exercise of a grassland dweller, and may predispose them to diseases of the musculoskeletal system and to digestive and behavioural disorders [5,28,29,30,31]. While it is considered an essential—however typically unmatched—welfare criterium for animals in human care to be able to express the full repertoire of behaviours observed in their wild conspecifics, wild animals also adapt their behaviour to suit environmental conditions [15,32]. Thus, while the need to provide domestic horses with species-appropriate living conditions is undisputed, the question arises whether changes in horses’ behaviour and corresponding time budgets may be appropriate adaptations to their environment and to which extent behavioural differences between domestic and feral horses can be used as a welfare indicator [10,14,15,33]. Given the disparity in environmental conditions, using wild activity budgets as the gold standard of welfare is flawed and fails to address the ambiguity of which behaviours are important for welfare and which are redundant in the context of a domestic horse [14]. Consequently, if equine management and welfare is to progress, objective and quantifiable comparisons between domestic environments and their influence on different equine demographics are needed to fully understand the impact of housing and management conditions and to establish evidence-based criteria to optimize horse welfare.

Traditionally, equine behavioural activity time budgets were measured by direct observation or manual video analysis, which was prone to observer bias, often limited to daylight hours and too time- and resource-intensive to be feasible for welfare assessment [13]. Recent technological advances in automated biotelemetry and sensor systems reduce the potential of a possible observer influence and enable accurate staff- and cost-efficient, non-invasive 24 h-time budget analysis over several days [8,34,35,36,37], which may facilitate their wide-spread adoption in welfare audits. As automated time budget analysis is a quantifiable and objective welfare assessment tool with limited qualitative resolution, the characterization of social interactions, different stereotypical and resting behaviours, requires supplementary methods if indicated.

Therefore, the present study aimed to establish and compare the activity time budgets of horses with chronic diseases and geriatric animals living in different housing systems and to determine if equine eating, resting and activity time budgets are affected by these management conditions.

## 2. Materials and Methods

### 2.1. Horses, Housing and Management Conditions

This prospective, observational cohort study was carried out in 104 horses: 54 warmbloods, 16 draft horses and 34 horses of other breeds, owned by an animal sanctuary. The horses were housed in neighbouring farms managed by equine sanctuary staff under similar conditions, but with three different management conditions and daily routines. Horses were either housed in (1) individual box stalls (16 m^2^) with paddock (27–67 m^2^/horse) or pasture (119–13,796 m^2^/horse) turn-out (farms 1 (*n* = 26) and 5 (*n* = 30), farm 3 (*n* = 9) in fall/winter); (2) small group (2–3 horses) stalls (8–12 m^2^/horse) with an attached pen (2–3 m^2^/horse) and paddock (68 m^2^/horse) or pasture (583 m^2^/horse) turn-out (farm 4, *n* = 28); or (3) open-air group housing on a paddock (40 m^2^/horse) or pasture (850 m^2^/horse) with a run-in shelter (lying surface 7 m^2^/horse) in group sizes of 9–10 horses (farm 2 (*n* = 11) and farm 3 (*n* = 9) in spring/summer) (Table S1). Box stalls and lying surfaces were bedded with straw (=edible bedding) or shavings. Stalls and paddocks were fully cleaned once daily and had additional manure removed as necessary. Every horse had either paddock turn-out for 3–24 h or pasture turn-out for 6–24 h daily, depending on weather and ground conditions. During hot summer temperatures, horses were turned-out to pasture at night. Horses had ad libitum access to clean water and were fed ad libitum hay during paddock turn-out with additional hay in the stable in the afternoon. Horses with poor dentition or additional nutritional requirements received supplementary feeding with hay cobs. Horses’ physical health and body condition score (BCS) were recorded by the same veterinarian prior to each tracking period [38]. Based on their age, physical and orthopaedic exam, horses were assigned to one of four health/age groups: (1) horses younger than 20 years with chronic orthopaedic diseases (chronic lameness > 1 (on the American Association of Equine Practitioner (AAEP) scale), *n* = 45); (2) geriatric horses (≥20 years) with chronic orthopaedic disease (*n* = 42); (3) sound (lameness ≤ 1) geriatric horses (*n* = 7); and (4) sound horses younger than 20 years (control group, *n* = 10).

### 2.2. Time Budget Analysis

**Figure 1:**
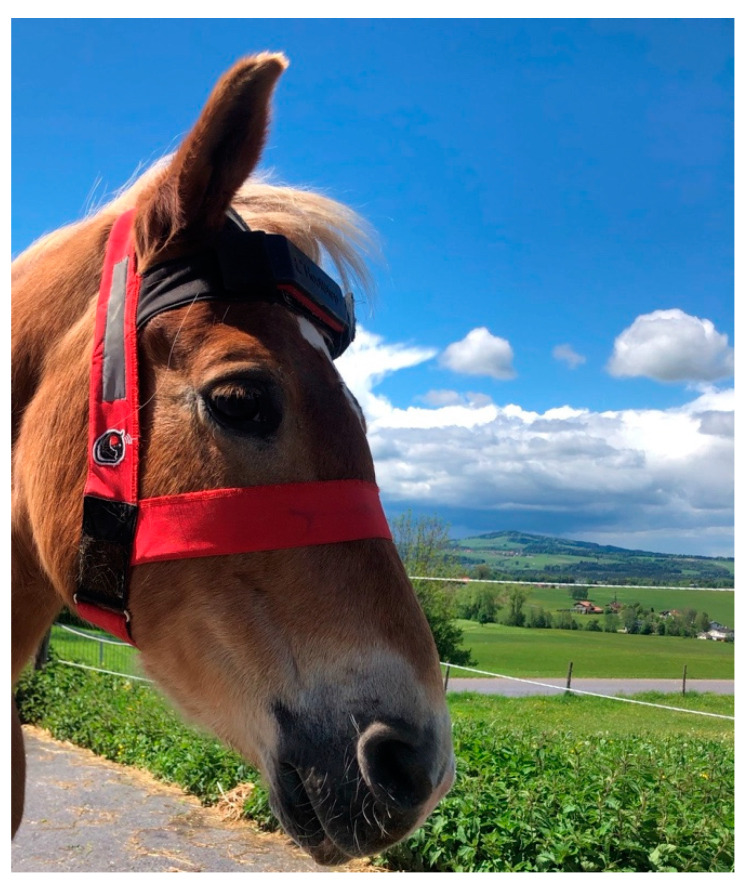
The wearable horse unit of the automated equine monitoring system (Hoofstep^®^) is attached to the horses’ forehead using a special flexible softshell head collar.

Horses were tracked twice within a 9-month period, once in spring/summer and once in fall/winter, for 5–10 days each using the Hoofstep^®^ automated equine monitoring system. Hoofstep’s wearable horse unit (96 mm × 47 mm × 45 mm, weight: 149 g) contains a GPS, an accelerometer, a gyroscope, and a radiotransmitter and is attached to the horses’ forehead using special flexible softshell head collars (Figure 1). During a pilot experiment, we determined that horses did not show any behavioural response to the wearable horse unit as they were all used to headcollars. Therefore, data recording started immediately following application of the device. The wearable horse units continuously record data, and can store the data for up to 24–72 h before sending it via mobile 3G network or by a WIFI connection to a farm unit, which then transfers the data to the cloud. Based on data generated by the accelerometer and the gyroscope and using an Artificial Intelligence algorithm, Hoofstep assigns a horse’s behaviour to one of four behavioural categories: (1) “feeding” (the time the horse is chewing—in any position or combination with other behaviours), (2) “resting” (without distinguishing between lying and standing), (3) “active” (slow locomotion, walk) and (4) “highly active” (fast movement (trot, canter) and potential stress behaviours such as headshaking). The variable “activity count (activity)” measures the transitions between behavioural categories per time period. Results are presented as percentage per hour and day in the corresponding Hoofstep^®^ app and provided in csv format for further analysis.

### 2.3. Statistical Analysis

As for linear models, causality needs to be assumed, the horse’s identity, housing (as factor), sex (gelding/mare), age, lameness (yes/no), and season were treated as explanatory variables. For an initial overall analysis of the mean time budget (Analysis of covariance, ANCOVA), target variables were mean activity time budgets per 24 h. These calculations were performed with the “R” statistical programming language (R Foundation for Statistical Computing, Vienna, Austria https://www.R-project.org/) [39]. In more detailed analyses of the daily time budgets, the effects of farm, turn-out condition, health/age group, and time of day were considered fixed effects in a series of multifactorial ANOVAs using NCSS 2020 Statistical Software (NCSS, LLC. Kaysville, UT, USA, ncss.com/software/ncss) and Graphpad Prism 9 (GraphPad Software, LLC. San Diego, CA, USA).

Additionally, descriptive statistics were calculated, e.g., pairwise correlations (Spearman) between age, gender, body condition score, edible bedding, extra food rations and time budgets. Intrasubject, intersubject, farm-, turn-out- and time-based variability (CV) were calculated by dividing the respective standard deviation with the corresponding mean (CV = s.d.*100/mean). For intraindividual CVs (CVg), the average of the individual CVs was computed. *p*-values of <0.05 were considered statistically significant.

### 2.4. Ethics Statement

This study was non-invasive and entailed only monitoring of the horses under their current conditions of life. No specific veterinary treatments or interventions were carried out for the purpose of this study. The study was carried out with approval by the Institutional Ethics Committee of the University of Veterinary Medicine Vienna (ETK-152/09/2019) in accordance with the “Good Scientific Practice. Ethics in Science und Research” guidelines implemented at the University of Veterinary Medicine Vienna and national legislation.

## 3. Results

### 3.1. Horses and Tracking

Of the 104 horses included in this study, 51 were mares and 53 geldings. Their age ranged from 2 to 32 years (mean: 19.88 years) and their body condition score from 2 to 8 (mean: 5.63) (Table S2). In health/age group 1 (lame, <20y), the horses’ mean age was 15.9 (±4 s.d.), in group 2 (lame, ≥20y) 25.1 (±3.4 s.d.), in group 3 (sound, ≥20y) 24.3 (±4 s.d.) and in group 4 (sound, <20y) 12.7 (±4.2 s.d.). All horses tolerated the wearable horse unit well, and no dermal irritations were observed. Data collection and transfer functioned well in all husbandry systems, and no technical problems were encountered.

### 3.2. Time Budgets and Activity Counts

**Figure 2:**
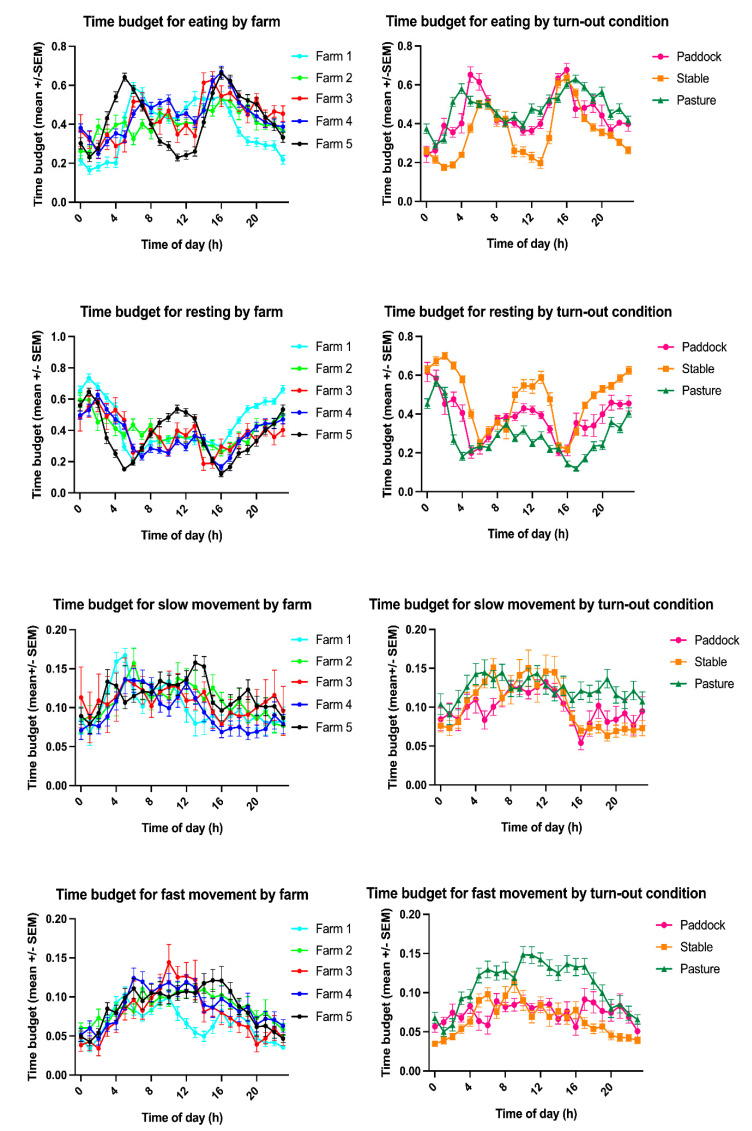
Time budgets for eating, resting, slow movement (“active”) and fast movement (“highly active”) by farm (**left**) and by turn-out condition (**right**) detailed by time of day.

**Figure 3:**
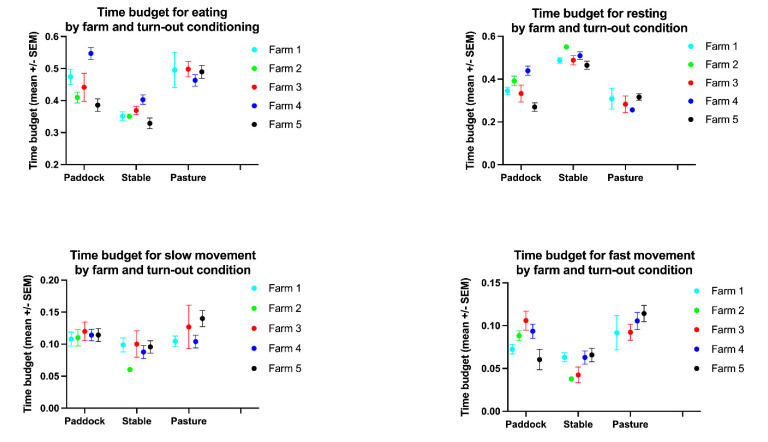
Time budgets for eating, resting, slow movement (“active”) and fast movement (“highly active”) by farm and turn-out condition.

**Figure 4:**
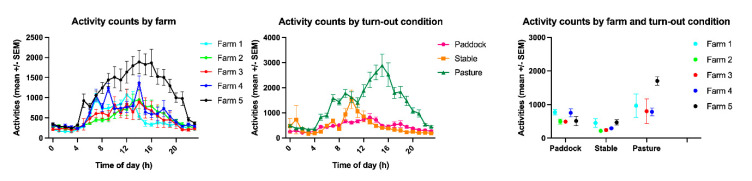
Activity counts by farm and turn-out condition detailed by time of day and by farm x turn-out condition.

The overall mean time budgets at the equine sanctuary were divided into 42% eating, 39% resting, 11% active and 8% highly active (Table 1). Time of day significantly affected all time budgets (*p* < 0.0001, Table 2, Table 3, Table 4 and Table S2) with eating peaking in the morning (6:00 a.m./7:00 a.m.) and in the afternoon (3:00 p.m.–5:00 p.m.) and resting predominantly at night between 9:00 p.m. and 4:00 a.m. (Figure 2). We note that, in farm 5, which most frequently employed turn-out overnight, resting occurred biphasic with a second peak around noon. Time budgets for locomotion and high activity as well as the activity count showed less pronounced temporal peaks but also significant differences over the course of the day with more activity during day-time hours and less at night (Table 2 and Table S3, Figure 2, Figure 3 and Figure 4). With the initial overall analysis of the mean daily time budget (ANCOVA), we generally observed significant influences of farm and season on the mean time budget (Table 3). The influence of the other explanatory variables was lower, with lameness influencing the eating, and age the fast movement time budget. In the more detailed analysis, for all time budgets and activity counts, the intraindividual variability was substantially lower than the interindividual variability (Table S4). Similarly, the variability within farms, turn-out conditions and time were substantially lower than between farms, turn-out conditions and time of day (Table S4).

**Figure 5:**
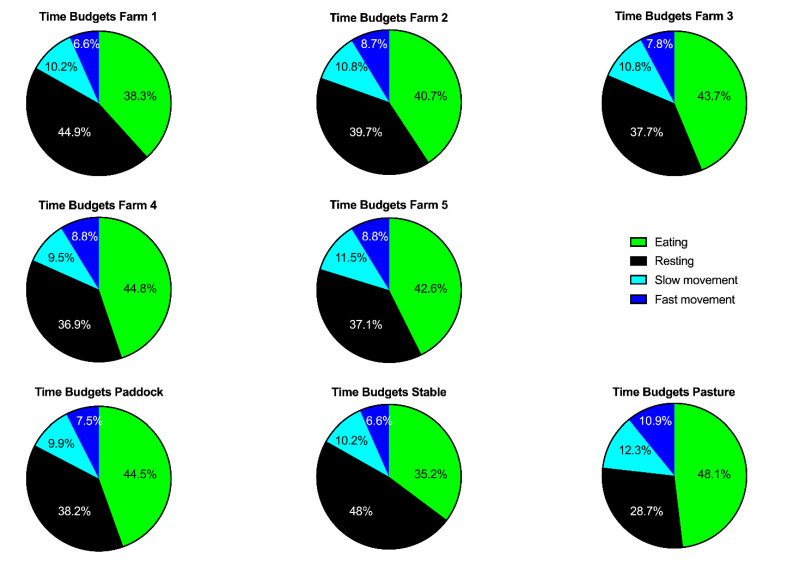
Pie charts illustrating the distribution of the time budgets by farm and turn-out condition.

The time budget for eating varied significantly between farms (*p* = 0.0004, Table 1, Table 3 and Table 4) and turn-out conditions (*p* < 0.0001, Figure 2, Figure 3 and Figure 5, Table 1 and Table 3) with farm accounting for 6.45% and turn-out for 21.61% of the total variance. The lowest mean time budget for eating was measured at Farm 1 with 38.3% (s.d. 13.8%), the highest in Farm 4 with 44.8% (s.d. 10.1%). Multiple comparison testing showed significant differences in the time budget for eating between paddock (mean 44.5% ± 11.3% s.d.) and stable (mean 35.2% ± 13.5% s.d., *p* < 0.0001), pasture (mean 48.1% ± 8.63% s.d.) and stable (*p* < 0.0001) but not between paddock and pasture (*p* = 0.4308).

The time budget for resting varied significantly between farms (*p* = 0.013) and turn-out conditions (*p* < 0.0001, Figure 2, Figure 3 and Figure 5, Table 1, Table 3 and Table 4) with farm accounting for 2.93% and turn-out for 40.43% of the total variance. The lowest mean time budget for rest was measured at Farm 4 with 36.87% (s.d. 11.94%), the highest in Farm 1 with 44.9% (s.d. 15.58%). Multiple comparison testing showed significant differences in the time budget for resting between paddock (mean 38.15% ± 10.77% s.d.) and stable (mean 47.97% ± 14.62% s.d., *p* < 0.0001), pasture (mean 28.7% ± 11.11% s.d.) and stable (*p* < 0.0001) and paddock and pasture (*p* = 0.0006).

The time budget for slow movement (“active”) varied significantly between turn-out conditions (*p* = 0.0056) but not between farms (*p* = 0.4798, Figure 2, Figure 3 and Figure 5, Table 1, Table 3 and Table 4) with turn-out accounting for 4.16% of the total variance. The lowest mean time budget for slow movement was measured at Farm 4 with 9.53% (s.d. 2.42%), the highest in Farm 5 with 11.5% (s.d. 2.03%). Multiple comparison testing showed significant differences between paddock (mean 9.87% ± 1.97% s.d.) and stable (mean 10.2% ± 3.16% s.d., *p* = 0.0472), pasture (mean 12.3% ± 1.44% s.d.) and stable (*p* = 0.0074) but not between paddock and pasture (*p* = 0.7074).

The time budget for fast movement (“highly active”) varied significantly between turn-out conditions (*p* < 0.0001) but not between farms (*p* = 0.6772, Figure 2, Figure 3 and Figure 5, Table 1, Table 3 and Table 4) with turn-out accounting for 13.25% of the total variance. The lowest mean time budget for fast movement was measured at Farm 1 with 6.64% (s.d. 2.06%), the highest in Farm 4 with 8.79% (s.d. 2.37%). Multiple comparison testing showed significant differences between paddock (mean 7.47% ± 1.2% s.d.) and stable (mean 6.6% ± 2.18% s.d., *p* = 0.0451), pasture (mean 10.9% ± 3.02% s.d.) and stable (*p* < 0.0001) and paddock and pasture (*p* = 0.0006).

The activity count varied significantly between farms (*p* = 0.0239) and turn-out conditions (*p* < 0.0001, Figure 4 and Figure 5, Table 1, Table 3 and Table 4) with farm accounting for 3.59% and turn-out for 21.33% of the total variance. The lowest mean activity count was measured at Farm 1 with 469 (s.d. 291), the highest in Farm 5 with 1085 (s.d. 578). Multiple comparison testing showed significant differences between paddock (mean 458 ± 174 s.d.) and stable (mean 483 ± 340 s.d., *p* = 0.0084), pasture (mean 1327 ± 784 s.d.) and stable (*p* < 0.0001) and paddock and pasture (*p* < 0.0001).

### 3.3. The Effect of Group, Age, Sex, BCS, Edible Bedding, Extra Food Rations and Season on Equine Time Budgets

**Figure 6:**
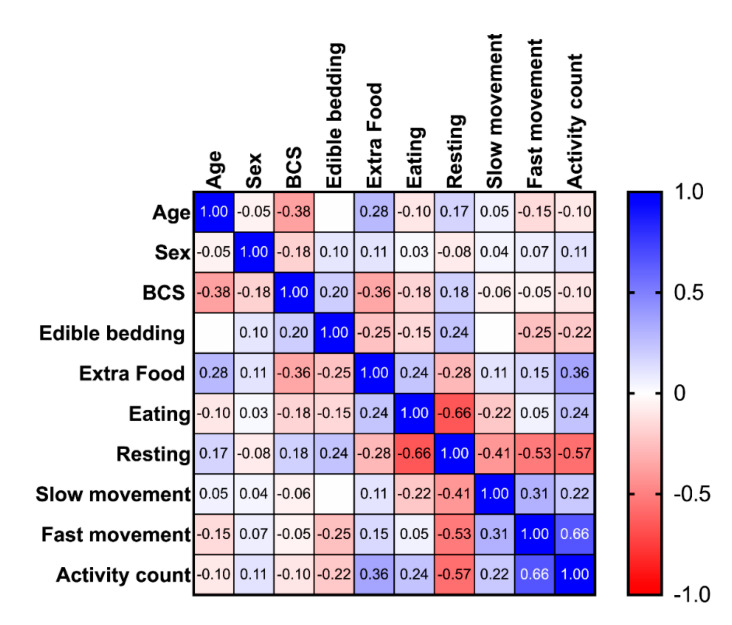
Heatmap of the Spearman correlation matrix for age, sex, body condition score (BCS), edible bedding, extra food, the time budget for eating, resting, slow movement and fast movement and the activity count.

Health/age group did not have a significant effect on any time budget or the activity count although age had a minimal effect on the time budget for slow movement and lameness on the eating time budget with the ANCOVA (Table 3 and Table 4). Age correlated negatively with BCS and positively, albeit with low correlation coefficients, with extra food and resting (see Table 5 for *p*- and r- values, Figure 6). BCS also correlated, in addition to age and sex, positively with edible bedding and resting and negatively with extra food and eating (Table 5). The availability of edible bedding and extra food correlated with each other and all time budgets except the time budget for slow movement (“active”). As expected, all time budgets (and the activity count), except eating and fast movement (“highly active”), correlated significantly with each other (Table 5). Furthermore, all time budgets and the activity count showed significant differences between tracking seasons (rounds, Table 3). For eating, the mean of round 1 (spring/summer) 42.84 (s.d. 8.98%) was significantly (*p* = 0.0024) higher than in round 2 (fall/winter) with 40.27% (s.d. 8.08%), while resting increased from 35.82% (s.d. 9.02%) to 43.6 5 (s.d. 8.48%, *p* < 0.0001). The three activity measures all were lower in fall/winter than in spring/summer, with active dropping from 11.8% (s.d. 6.3%) to 9.99% (s.d. 5%, *p* = 0.0366), highly active from 9.53% (s.d. 4.5%) to 6.14% (s.d. 3.8%, *p* < 0.0001) and the activity count from 887.2 (s.d. 633.8) to 326.2 (s.d. 182.9, *p* < 0.0001).

## 4. Discussion

Horses in this study were eating 42% of their day, which is within the wide range of 10–64% measured in domestic horses but below the 50.82–66.6% reported for semi-feral horses [13,16,23,24,25,26,36,40,41,42,43,44,45]. Coinciding with the literature reporting 60–70% day-time and 30–40% night-time feeding [46], eating times were highest in the morning and the afternoon and lowest in the night and very early morning hours, even in horses that were turned out on pasture overnight during high summer temperatures. Notably, in horses with restricted access to forage (farms 1 and 5, during the fall tracking period also farms 3 and 4) eating peaked immediately after feeding. We recorded the highest eating time for horses on pasture (mean 48.1% ± 8.63% s.d.) and the lowest for stabled horses (mean 35.2% ± 13.5%), which may be due to stabling occurring predominantly overnight (except during heat periods) when horses eat the least, lower food availability in the stable, palatability of grass versus hay and the variable caloric density of the accessible food. Given the caloric requirements of 16.7 Mcal/day/500 kg horse at rest and the average caloric density and dry matter (DM) proportion of pasture (2.23 Mcal/kg DM, 30% DM) and grass hay (1.78 Mcal/kg DM, 90% DM), a 500 kg horse needs to consume 25 kg grass or 10.3 kg hay, with correspondingly different required feeding times, to cover its caloric requirements [47,48,49,50]. However, food intake is controlled not just by energy-related homeostatic signals but also somatosensory and motivational stimuli, explaining why diet has the greatest effect on equine time budgets [10,51]. Indeed, the incidence of stereotypic behaviour increases with decreasing access to roughage, non-edible bedding and corresponding lower feeding times [2,10,29,41,44,46,51,52,53,54]. Furthermore, as the equine digestive tract has adapted to a continuous intake of fibrous low energy herbage, restricted access to roughage, may induce health problems such as gastric ulceration, constipation or dysfermentation [9,29,41,42,46]. Accordingly, feed intake pauses of less than 4 h are recommended to avoid compromises in animal welfare [46]. Even stabled horses which are fed ad libitum, divide their feed into approximately 10 meals, comparable to their free-ranging conspecifics and do not pause voluntarily for longer than 3 to 4 h between meals [20,46,51,52,55,56,57,58]. Although feeding hay from the ground would be most natural and a position favoured by horses, the use of medium-sized haynets can slow the feed intake rate by 25% and may be helpful in extending feeding times in horses that cannot get ad libitum roughage [44,50,51,52]. The equine sanctuary implemented haynets for the evening rations for stabled horses in response to the relatively low eating time budgets observed in this study in stabled horses, which indeed increased eating time budgets during a follow-up tracking period (data not shown).

The overall time budget for resting of 39%, which included periods of inactivity and sleep, was higher compared to free-ranging conspecifics (12.9–29.3%) but well within the 15.6–66% range reported for domestic horses [13,16,23,24,25,26,36,40,41,42,43,44,45]. As horses divide their day mostly between eating and resting behaviour, the time budget for resting expectedly correlated negatively with eating (*p* < 0.0001, r = 0–0.655) and thus was highest in stabled (mean 47.97% ± 14.62% s.d.) and lowest in pastured (mean 28.7% ± 11.11% s.d.) horses. In accordance with the literature [3,5,23,26,28,45,59,60,61,62,63,64], resting peaked at night between 9:00 p.m. and 4:00 p.m. regardless of turn-out and management conditions. Resting behaviour is a reliable indicator for equine welfare, with horses living under inappropriate environmental conditions showing decreased resting times [7,17,21,59,65]. The tracking device used in this study could not discern between standing and lying, thus limiting the interpretation of the observed resting behaviour. Given the importance of rapid eye movement (REM) sleep, which only occurs in recumbent horses, for many physiological and cognitive functions, future studies examining the lying behaviour of geriatric or orthopaedically challenged horses are needed to further examine this important aspect of equine welfare. Furthermore, as the present study quantified time budgets and did not qualitatively assess the horses’ behaviour, further studies evaluating stereotypical behaviours and social interactions under different husbandry conditions are essential to fully assess their welfare implications.

Surprisingly, considering the geriatric and orthopedically challenged equine demographic, the mean overall time budget for movement of 19% in this study is on the upper end of the reported 4.1–19.1% range for domestic horses and higher than the 4.3–13.4% observed in free-ranging conspecifics [13,16,23,24,25,26,36,40,41,42,43,44,45]. The time budgets for movement can only be compared with caution due to differences in methodology, the low number of studies measuring the 24 h movement time budget in horses and the unclear distinction between foraging and movement in the few studies that did [1,13,66]. In this study, movement is divided into 11% slow movement (“active”, mean 11% ± 12.7% s.d.), which corresponds to walk, the main type of locomotion observed in free-ranging horses and 8% fast movement (“highly active”, mean 8% ± 10.2% s.d.), which includes trot and canter but also other movements such as headshaking (e.g., to ward off flies) or stereotypical behaviour. In this context, the activity counts may help to identify stress or stereotypical behaviour by highlighting frequent changes in behaviour as a potential warning sign for poor welfare.

The time budget for movement is influenced by different stabling conditions and feeding schedules and considered a reliable indicator of equine welfare [6,17,21,28,59,65,67,68,69,70,71,72,73]. Horses living under inappropriate environmental conditions, including insufficient forage opportunities, high stocking densities and small enclosures, increased their active locomotion patterns [6,17,21,28,59,65,67,68,69,70,71,72,73]. In addition, domestic mares kept in stalls for 72 h exhibited higher levels of movement during subsequent turn-out than mares kept on pasture with conspecifics [6,72]. Interestingly, in the present study, the combined movement time budget (“active” + “highly active”) was highest in horses on pasture (22%), closely followed by horses in a paddock (19.7%) and then in a stable (14.3%). This may be partly due to compensatory movement following confinement upon release onto pasture or warding off pests but should be investigated further, as to date no other studies reported the movement time budget of pastured domestic horses. The significant difference in movement time budgets and activity count between seasons (tracking rounds), with substantially higher values during the summer months, supports the association between movement counts and pest defence, which was also observed in free-ranging Camargue horses during the summer [24]. In accordance with the literature [6,21,28,59,65,67,68,69,70,71,72,73], movement in the present study occurred mostly during day-time hours and least at night; however, activity peaks could be observed just prior to morning feeding in horses kept on restricted feeding and non-edible bedding (farm 1), which may be linked to anticipatory behaviour or stressful feeding times [57,58].

The geriatric horses and horses suffering from chronic orthopaedic disease had time budgets equivalent to the healthy control group and largely also within the ranges observed in free-ranging conspecifics, demonstrating that being in appropriate living conditions age and/or orthopaedic disease does not significantly affect equine behaviour time budgets. Monitoring the horses in the different farms in different seasons using the automated tracking system allowed us to establish and compare the time budgets of the different farms at different time points and identify potential areas for improvement, such as the provision of hay nets in stabled horses overnight. The effects of husbandry practices on equine health and welfare are well established [1,3,5,7,8,17,32]. In particular, spatial restrictions, lack of social contact and species-inappropriate roughage access and foraging opportunities may contribute to musculoskeletal and gastrointestinal diseases and behavioural disorders [1,3,5,7,8,11,17]. Indeed, management changes, such as ad libitum feeding or the use of hay nets to extend feeding time, can reduce stereotypical behaviours [1,3,5,7,8,17,74]. In the present study, horses living in open-air group housing on a paddock (farm 2) had a more uniform temporal distribution of feeding and movement with less pronounced peaks compared to horses living in more restricted husbandry systems, which may be associated with less stress and better accommodation of equine gastrointestinal physiology, thus contributing to horses’ welfare. Although there were significant differences in the eating and resting time budgets and the activity counts between farms, these differences were small, e.g., for eating, it ranged between 38.3–44.8%, and manifested predominantly in the more or less homogenous distribution throughout the day. The small range of time budgets observed between the different housing systems in this study compared to the substantial ranges reported in the literature is not surprising as all farms are under the management of the same equine sanctuary, which actively attempts to provide horses with sufficient foraging, exercise opportunities and professional care.

## 5. Conclusions

**Figure 7:**
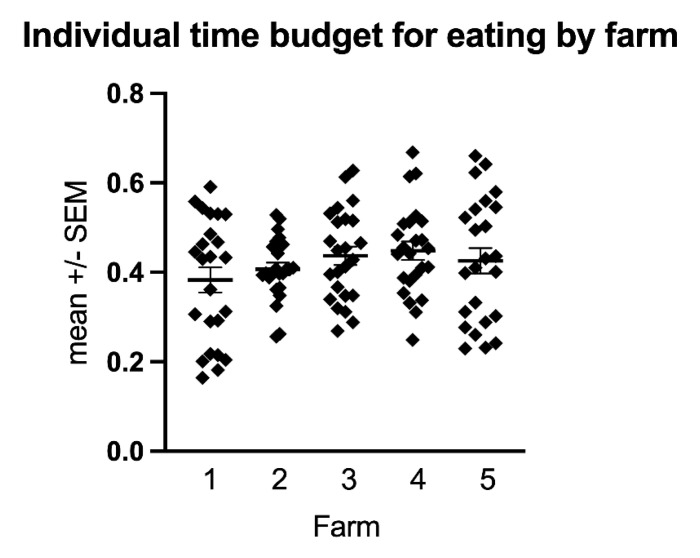
The scatter plot shows the individual mean time budget for eating for all horses included in this study divided by farm. For each farm, the mean ± SEM is indicated. The time budget for eating is similar for most horses except for a few outliers, which might be an indication for potential health or welfare issues.

As welfare is defined as an animal’s ability to cope with the environment it finds itself in, welfare optimization strategies should be based on establishing and pursuing highly-motivated behaviours and corresponding time budgets that contribute to equine welfare and well-being in their specific environment rather than blindly pursuing wild-like behaviour as the gold standard [14,75,76]. To facilitate comparison between husbandry systems and evaluate the success of interventions aimed at improving equine welfare, objective and quantifiable parameters and standardized measurement methods are needed, which can be applied consistently by different researchers; time budgets measured by automated tracking tools are uniquely suited for this purpose. Indeed, the automated tracking system utilized in this study allowed standardized and observer-independent measurement and comparison of the behaviour time budgets of horses in different husbandry practices. We could demonstrate that geriatric horses and horses suffering from chronic orthopaedic disease can, under appropriate husbandry conditions, exhibit behaviour time budgets equivalent to healthy adult controls. While similar time budgets do not imply good welfare per se, they indicate an equal ability of the geriatric and chronically lame horses compared with the healthy control group to cope with their environment. Time budget analysis revealed significant differences between farms and turn-out conditions and could identify potential areas for improvement. The more uniform temporal distribution of feeding and movement of horses living in open-air group housing, compared to horses living in more restricted husbandry systems, may indicate less stress and may provide an additional informative way of analysing time-budget studies in the context of welfare assessment. As a future perspective, outliers from the mean time budgets measured under specific husbandry conditions (Figure 7) may help to identify individual horses that may be at risk of poor welfare and require additional qualitative assessment to infer their welfare status.

## Figures and Tables

**Table 1:** Time budget for eating, resting, slow movement (“active”), fast movement (“highly active”) and the activity counts by farm, season and turn-out condition.

Farm	Month	Turn-out	Time Budget for Eating					Time Budget for Resting					Time Budget for Slow Movement					Time Budget for Fast Movement					Activity Count				
			Mean	s.d.	95% Conf. Int.			Mean	s.d.	95% Conf. Int.			Mean	s.d.	95% Conf. Int.			Mean	s.d.	95% Conf. Int.			Mean	s.d.	95% Conf. Int.		
Overall			42.0	29.0	42.3	-	41.6	39.1	29.3	39.4	-	38.7	11.0	12.7	11.1	-	10.8	8.0	10.2	8.1	-	7.9	684.6	2531.7	716.3	-	652.9
1	Overall		38.7	29.1	39.3	-	38.1	44.4	28.7	45.0	-	43.8	10.3	12.4	10.5	-	10.0	6.7	9.6	6.9	-	6.4	529.6	3721.9	610.0	-	449.2
	May- June	Paddock	47.5	28.1	48.9	-	46.1	33.6	25.6	34.9	-	32.3	10.9	12.8	11.5	-	10.2	8.0	9.6	8.5	-	7.5	873.6	2098.9	978.6	-	768.5
		Stable	35.9	28.9	36.8	-	35.0	47.5	29.3	48.4	-	46.6	10.0	12.9	10.4	-	9.6	6.6	9.6	6.9	-	6.3	492.2	5105.5	650.4	-	334.0
		Pasture				-					-					-					-					-	
	October-November	Paddock	43.5	30.3	45.9	-	41.1	39.1	27.1	41.2	-	36.9	10.9	11.9	11.9	-	10.0	6.5	9.4	7.2	-	5.7	525.3	1099.7	611.8	-	438.8
		Stable	34.5	27.9	35.8	-	33.2	50.0	27.3	51.2	-	48.7	10.1	11.5	10.6	-	9.6	5.4	9.0	5.8	-	5.0	255.6	355.1	271.8	-	239.4
		Pasture	50.2	30.2	54.2	-	46.3	30.3	26.2	33.8	-	26.9	10.4	11.2	11.9	-	8.9	9.1	12.2	10.7	-	7.5	1121.5	2672.9	1472.3	-	770.6
2	Overall		41.8	26.0	42.6	-	41.0	38.0	26.9	38.9	-	37.2	11.3	12.4	11.6	-	10.9	8.9	9.9	9.2	-	8.6	515.1	826.1	541.1	-	489.1
	June	Paddock	41.8	26.0	42.7	-	40.8	38.3	26.7	39.3	-	37.3	11.0	11.8	11.4	-	10.6	9.0	9.9	9.3	-	8.6	641.0	988.3	679.6	-	602.5
		Stable	33.4	24.6	39.6	-	27.2	56.3	27.2	63.2	-	49.5	6.1	10.6	8.8	-	3.5	4.1	7.2	5.9	-	2.3	227.0	183.4	273.0	-	181.0
		Pasture				-					-					-					-					-	
	November	Paddock	42.7	25.9	44.1	-	41.2	35.8	26.7	37.3	-	34.3	12.4	13.6	13.1	-	11.6	9.1	10.1	9.7	-	8.6	284.5	212.1	296.4	-	272.6
		Stable	36.9	25.3	43.4	-	30.4	53.8	27.4	60.9	-	46.7	5.9	10.9	8.7	-	3.1	3.4	5.8	4.9	-	1.9	202.6	133.4	237.0	-	168.3
		Pasture				-					-					-					-					-	
3	Overall		44.3	28.8	45.5	-	43.1	35.2	28.5	36.4	-	34.0	12.4	13.7	13.0	-	11.9	8.0	9.6	8.4	-	7.6	436.4	677.8	473.0	-	399.9
	August	Paddock	49.2	34.4	58.7	-	39.6	29.2	27.3	36.8	-	21.6	10.3	9.0	12.8	-	7.8	11.3	14.4	15.3	-	7.3	368.9	235.3	434.2	-	303.7
		Stable	31.8	26.8	36.9	-	26.7	55.3	29.0	60.7	-	49.8	10.4	10.8	12.4	-	8.3	2.6	5.2	3.6	-	1.6	156.6	145.6	184.2	-	129.0
		Pasture	49.0	28.5	50.6	-	47.4	28.2	26.3	29.7	-	26.7	13.7	15.3	14.5	-	12.8	9.2	9.1	9.7	-	8.6	738.6	1056.8	845.0	-	632.2
	November	Paddock	41.6	26.8	45.3	-	38.0	33.6	22.9	36.7	-	30.5	13.2	10.6	14.6	-	11.8	11.6	12.7	13.3	-	9.8	487.8	388.8	540.5	-	435.1
		Stable	37.2	27.6	39.5	-	35.0	47.5	28.8	49.9	-	45.2	10.1	11.2	11.0	-	9.2	5.1	8.1	5.8	-	4.4	276.8	394.3	309.0	-	244.6
		Pasture				-					-					-					-					-	
4	Overall		44.8	27.7	45.6	-	44.0	36.7	28.0	37.5	-	35.9	9.8	12.1	10.2	-	9.5	8.7	11.2	9.0	-	8.4	595.3	1120.2	627.1	-	563.6
	September	Paddock				-					-					-					-					-	
		Stable	43.1	26.3	45.5	-	40.7	41.3	26.5	43.7	-	38.9	8.7	11.0	9.7	-	7.7	6.9	8.7	7.7	-	6.1	396.1	416.5	434.4	-	357.8
		Pasture	46.0	27.6	47.1	-	44.8	32.9	27.7	34.1	-	31.8	10.5	12.3	11.0	-	10.0	10.6	11.9	11.1	-	10.1	788.8	1345.2	843.8	-	733.7
	January	Paddock	53.8	26.7	56.1	-	51.5	24.4	23.1	26.3	-	22.5	11.8	12.0	12.8	-	10.8	10.0	11.9	11.0	-	9.0	878.6	1507.8	1006.1	-	751.2
		Stable	40.3	27.7	41.7	-	38.9	45.3	27.9	46.7	-	43.9	8.4	11.8	9.0	-	7.8	6.0	9.8	6.5	-	5.5	261.8	414.9	282.7	-	240.9
		Pasture																									
5	Overall		43.3	31.3	44.1	-	42.5	36.0	31.9	36.8	-	35.2	12.1	13.2	12.4	-	11.7	8.7	10.6	8.9	-	8.4	1110.8	2265.1	1166.7	-	1054.9
	July- August	Paddock	35.8	28.5	44.2	-	27.3	30.1	27.1	38.1	-	22.2	19.5	16.5	24.4	-	14.6	14.6	9.4	17.4	-	11.8	1574.9	1751.2	2092.4	-	1057.5
		Stable	26.2	25.0	28.1	-	24.3	49.1	30.4	51.4	-	46.8	14.8	15.6	16.0	-	13.6	9.9	11.5	10.8	-	9.0	841.7	1893.0	986.5	-	697.0
		Pasture	50.1	28.4	51.1	-	49.2	25.6	27.8	26.6	-	24.7	13.5	13.0	13.9	-	13.0	10.8	10.7	11.1	-	10.4	1702.0	2783.6	1795.3	-	1608.6
	December	Paddock	38.5	35.4	40.9	-	36.2	44.9	33.2	47.0	-	42.7	11.1	12.4	11.9	-	10.2	5.6	9.7	6.2	-	4.9	351.4	770.8	402.2	-	300.6
		Stable	37.5	33.6	39.3	-	35.7	50.6	31.8	52.3	-	48.9	7.5	11.7	8.1	-	6.8	4.4	8.3	4.9	-	4.0	200.0	199.2	210.8	-	189.2
		Pasture				-					-					-					-					-	

**Table 2:** Time budgets for eating, resting, slow movement (“active”) and fast movement (“highly active”) and the activity count by time of day.

	Time Budget for Eating (%)		Time Budget for Resting (%)		Time Budget for Slow Movement (%)		Time Budget for Fast Movement (%)		Activity Count	
	Mean	s.d.	Mean	s.d.	Mean	s.d.	Mean	s.d.	Mean	s.d.
00:00	25.39	23.23	45.31	25.07	8.38	11.14	4.26	7.07	340.33	2201.22
01:00	23.30	22.04	48.74	23.79	7.44	11.20	3.85	6.05	336.82	2458.94
02:00	23.74	22.13	46.61	24.22	8.64	11.36	4.35	6.54	237.46	898.87
03:00	27.70	22.56	40.20	24.34	9.77	11.59	5.66	7.82	180.47	230.20
04:00	29.87	21.17	35.61	23.02	11.18	11.74	6.67	8.47	206.21	231.46
05:00	35.91	20.59	28.04	19.22	11.66	11.12	7.73	8.44	368.12	489.36
06:00	41.78	21.57	22.23	18.52	11.35	11.43	7.98	8.78	467.32	621.99
07:00	41.35	23.27	23.66	21.49	10.37	10.27	7.95	9.05	685.41	778.00
08:00	35.07	23.46	30.10	22.46	10.10	9.83	8.07	8.28	634.36	800.58
09:00	35.67	22.32	28.38	21.99	10.47	10.33	8.81	8.81	739.46	970.91
10:00	35.45	21.67	28.02	20.62	10.35	9.65	9.52	9.76	730.83	1222.96
11:00	31.19	21.45	32.13	22.04	11.02	10.08	9.00	8.78	714.23	1118.62
12:00	33.23	21.71	30.84	21.55	10.43	9.79	8.83	8.29	827.57	1462.30
13:00	32.09	21.83	32.26	21.47	10.25	10.39	8.74	8.84	832.82	1690.96
14:00	41.83	22.40	24.53	20.52	9.47	10.11	7.50	8.25	878.74	1364.45
15:00	46.91	22.96	20.57	18.76	8.33	9.51	7.52	9.09	715.40	1325.86
16:00	49.95	20.72	18.10	17.11	7.54	8.77	7.75	9.68	723.97	1549.86
17:00	46.54	21.04	21.60	19.01	8.03	9.76	7.16	8.46	614.44	1008.52
18:00	40.30	21.49	28.16	21.41	8.30	9.75	6.58	8.30	627.81	902.45
19:00	37.85	22.14	31.11	22.61	8.06	10.07	6.31	8.67	517.78	735.37
20:00	36.72	22.55	33.75	23.49	8.07	10.15	4.79	7.11	423.57	508.00
21:00	32.37	22.37	37.77	22.80	8.27	11.01	4.93	6.78	359.12	446.76
22:00	33.05	22.32	37.09	23.31	8.34	10.56	4.85	7.06	258.25	293.40
23:00	29.69	22.22	41.91	23.92	7.65	10.29	4.07	6.13	230.49	218.50

**Table 3:** ANCOVA results: Sums of squares and significance (* *p* < 0.05, ** *p* < 0.01, *** *p* < 0.001) of the explanatory variables (farm, sex, age, presence/absence of orthopaedic disease (lame), season and horse) and adjusted R^2^ for the ANCOVAs for all time budgets.

Time Budget	Farm	Sex	Age	Lame	Season	Horse	R^2^
Eating	0.09 ***	0.01	0.00	0.01 *	0.04 ***	0.90 ***	0.61
Resting	0.15 ***	0.00	0.00	0.01	0.27 ***	0.66	0.26
Active	0.01	0.01	0.00	0.00	0.01 *	0.31	0.10
H Act.	0.01 **	0.00	0.01 **	0.00	0.05 ***	0.21 ***	0.20

**Table 4:** Results of ANOVA: *p*-values, Dfn, Dfd and F values for the comparison by farm, turn-out condition, time of day and health/age group are listed for all time budgets and the activity count.

		Farm	Turn-Out	Time of Day	Health/Age Group
Eating	*p*-value	0.0004	0.0001	0.0001	0.09
	Dfn	4	2	23	3
	Dfd	240	240	2298	131
	F	5.332	35.7	44.7	2.21
Resting	*p*-value	0.013	0.001	0.0001	0.106
	Dfn	4	2	23	3
	Dfd	251	251	2298	131
	F	3.23	89.3	71.78	2.077
Slow movement	*p*-value	0.4798	0.0056	0.0001	0.925
	Dfn	2	4	23	3
	Dfd	240	240	2298	131
	F	5.293	0.8746	11.03	0.157
Fast movement	*p*-value	0.6772	0.0001	0.0001	0.601
	Dfn	4	4	23	3
	Dfd	240	240	2298	131
	F	0.5802	18.6	24.36	0.624
Activity Count	*p*-value	0.0239	0.0001	0.0001	0.504
	Dfn	2	4	23	3
	Dfd	236	236	2272	129
	F	34.1	2869	22.78	0.785

**Table 5:** The correlation coefficients (r) for age, sex, body condition score (BCS), edible bedding, extra food, and the time budgets for eating, resting, active, highly active and the activity counts (bold indicates significance, *** *p* < 0.001, ** *p* < 0.01, * *p* < 0.05).

	Age	Sex	BCS	Edible Bedding	Extra Food	Eating	Resting	Slow Movement	Fast Movement	Activity Count
Age	1	−0.05	−0.38 ***	0	0.28 ***	−0.1	0.17 *	0.05	−0.15 *	−0.1
Sex	−0.05	1	−0.18 *	0.1	0.11	0.03	−0.08	0.04	0.07	0.11
BCS	−0.38 ***	−0.18 *	1	0.2 **	−0.36 ***	−0.18 **	0.18 *	−0.06	−0.05	−0.1
Edible Bedding	0	0.1	0.2 **	1	−0.25 ***	−0.15 *	0.24 **	0	−0.25 ***	−0.22 **
Extra Food	0.28 ***	0.11	−0.36 ***	−0.25 ***	1	0.24 **	−0.28 ***	0.11	0.15 *	0.36 ***
Eating	−0.1	0.03	−0.18 **	−0.15 *	0.24 **	1	−0.66 ***	−0.22 **	0.05	0.24 **
Resting	0.17 *	−0.08	0.18 *	0.24 **	−0.28 ***	−0.66 ***	1	−0.41 ***	−0.53 ***	−0.57 ***
Slow Movement	0.05	0.04	−0.06	0	0.11	−0.22 **	−0.41 ***	1	0.31 ***	0.22 **
Fast Movement	−0.15 *	0.07	−0.05	−0.25 ***	0.15 *	0.05	−0.53 ***	0.31 ***	1	0.66 ***
Activity count	−0.1	0.11	−0.1	−0.22 **	0.36 ***	0.24 **	−0.57 ***	0.22 **	0.66 ***	1

## Data Availability

Data are available from the authors upon reasonable request.

## Supplementary Materials

The following are available online at https://www.mdpi.com/article/10.3390/ani11071867/s1. Table S1: Details of the different farms’ housing and turn-out characteristics. Table S2: List of all horses included in this study, detailing their age, sex, breed and time budgets by tracking period (round) and whether they were stabled on edible bedding and received additional food. Table S3: The time budgets (mean ± s.d.) are detailed by farm, tracking period (round) and time of the day. Table S4: The CVg (=intra-individual variability; for horse: intraindividual, for farm/turn-out condition/time of day: within the same farm/turn-out condition/time) and Cvi (=inter-individual variability; for horse: between horses, for farm/turn-out condition/time of day: between farms/turn-out conditions/times) are detailed for the time budgets and activity counts by horse, farm, turn-out condition and time of day.
